# Nursery Government in Its Sanitary Aspects

**Published:** 1856-04

**Authors:** T. Herbert Barker


### NURSERY GOVERNMENT IN ITS SANITARY ASPECTS.

By T. HERBERT BARKER, M.D., F.R.C.S.

Among all the physical evils now occupying the attention of men who devote their studies to the question of Public Health, there can be none more important than the excessive mortality of infants which marks our present stage of civilisation. When we read—not in the coloured statements of a declamatory writer, but in the Report of the Registrar-General*—that, in the space of seven years, in the city of Manchester alone, "thirteen thousand three hundred and sixty-two children perished, over and above the mortality

* For the year 1849.

natural to mankind", we feel compelled to investigate the causes of such an enormous sacrifice of infant life. Some of these causes are doubtless peculiar to that dense centre of our manufacturing population; but that there are other and more general causes, not confined to any particular localities, but everywhere contributing to swell the sum of infant mortality, will be clearly shown by the following tables of graveyard statistics.

In order that we may see in their true significance the facts of the case, it is proper to observe, in the first place, that our infantile population is entirely free from many of the evils which cause excessive mortality among adults. Intemperance, dissipation, over-work and anxiety, the fatal accidents so frequent in several perilous occupations—these, and many other causes of a preternatural ratio of adult mortality, must be entirely left out of our calculations on the annual sacrifice of infant life. In other words, according to the ratio by which these causes make an *increase* of adult mortality, they should show a *decrease* in the mortality of infants. But our tables will show that the special causes of mortality in infancy and childhood are either so numerous or so potent, that they are found sufficient, not only to counterbalance the considerable sum of circumstances favourable to infant lives, but also to raise their average mortality far above that of adults! These remarks may serve to explain the assertion, that the *proportion* of deaths in infancy to those in adult age is, when all circumstances are considered, even greater than the following tables show! For if A. ought to have been a gainer to the amount of four, but finds himself a loser to the amount of four, it follows that he has lost eight. Just so, these tables show that our infant population *loses* more lives within the first five years, than our adult population, in any subsequent average period of five years, in the ratio of nearly twelve to one; but it must also be considered, that it ought to have *gained* by the exemptions and advantages to which we have alluded. Consequently, the value of these exemptions must be *added* to the sum of excessive infantine mortality, in order to give the total loss.

The total disproportionate mortality of infancy has never been exhibited in a strict statistical form. Yet the following Tables, compiled from the returns of a strictly rural district (Bedford), from the General Reports for England and Wales, and from the returns of various continental states, shew that we have ample grounds for a suspicion that serious errors are prevalent in the management of infancy.

### TABLE I.

*Number of Deaths at different ages in Bedford during sixteen years, from* 1837 *to* 1853.

| Sex. | 6 months. | 1 year. | 2 years. | 3 years. | 4 years. | 5 years. | 5 to 25 years. | 25 to 45 years. | 45 to 65 years | 65 to 85 years. | 85 to 100 years. | Total. | Deaths under 5 years in 1000 at all ages. |
|---|---|---|---|---|---|---|---|---|---|---|---|---|---|
| Males ...... | 398 | 202 | 90 | 48 | 37 | 65 | 327 | 373 | 351 | 348 | 17 | 2256 | 372 |
| Females .... | 284 | 184 | 101 | 44 | 35 | 57 | 380 | 375 | 352 | 388 | 23 | 2223 | 317 |
| Totals .... | 682 | 386 | 191 | 92 | 72 | 122 | 707 | 748 | 703 | 736 | 40 | 4479 | 345 |

### TABLE II.

*Number of Deaths at various ages in fourteen rural parishes near Bedford, during sixteen years, from* 1837 *to* 1853.

| Sex. | 6 months. | 1 year. | 2 years. | 3 years. | 4 years. | 5 years. | 5 to 25 years. | 25 to 45 years. | 45 to 65 years. | 65 to 85 years. | 85 to 100 years. | Total. | Deaths under 5 years in 1000 at all ages. |
|---|---|---|---|---|---|---|---|---|---|---|---|---|---|
| Males ...... | 334 | 149 | 60 | 25 | 19 | 44 | 174 | 118 | 154 | 267 | 17 | 1361 | 464 |
| Females .... | 235 | 120 | 64 | 28 | 29 | 57 | 336 | 197 | 214 | 265 | 12 | 1557 | 335 |
| Totals .... | 569 | 269 | 124 | 53 | 48 | 101 | 510 | 315 | 368 | 532 | 29 | 2918 | 399 |

TABLE III.

*Number of Deaths in Infancy and Childhood, as compared with the Number of Deaths occurring at all ages, in England and Wales.*

| Date. | Under 1 year. | From 1 to 5 years. | From 5 to 10 years. | All other ages. | Totals. | Deaths under 5 yrs. in 1000 at all ages. |
|---|---|---|---|---|---|---|
| 1838 | 73,606 | 58,531 | 16,138 | 194,485 | 342,760 | 385 |
| 1839 | 74,531 | 62,166 | 16,715 | 185,566 | 338,978 | 403 |
| 1840 | 77,411 | 67,909 | 20,207 | 194,360 | 359,687 | 404 |
| 1841 | 74,210 | 59,373 | 17,868 | 192,396 | 343,847 | 388 |
| 1842 | 78,704 | 60,331 | 17,208 | 193,276 | 349,519 | 397 |
| 1843 | 79,253 | 58,370 | 16,142 | 192,680 | 346,445 | 397 |
| 1844 | 80,086 | 59,918 | 17,371 | 199,558 | 356,933 | 392 |
| 1845 | 77,426 | 58,151 | 15,852 | 197,397 | 349,366 | 388 |
| 1846 | 93,644 | 66,976 | 16,190 | 213,605 | 390,315 | 411 |
| 1847 | 88,508 | 69,863 | 19,120 | 242,175 | 419,666 | 379 |
| Total | 797,379 | 621,588 | 172,811 | 2,005,498 | 3,597,516 | 394 |

TABLE IV.

*Number of Deaths in Infancy and Childhood, as compared with the Number of Deaths at all ages, in various Continental States.*

| Country. | Date of statistics. | Under 1 year. | From 1 to 5 years. | From 5 to 10 years. | All other ages. | Totals. | Deaths under 5 years in 1000 at all ages. |
|---|---|---|---|---|---|---|---|
| France ...... | Average 1817-31 | 176,708 | 108,609 | 37,312 | 361,900 | 784,529 | 363 |
| Prussia ...... | 1839-41 | 103,509 | 74,937 | 19,443 | 197,914 | 399,136 | 447 |
| Sweden ...... | 1806-35 | 15,540 | 8,513 | 2,583 | 39,939 | 66,576 | 361 |
| Norway ...... | 1801-35 | birth to | 10 years | 7,867 | 13,326 | 21,193 | 371 |
| Russia ...... | 1825-27 | bth.to 5 | 314,969 | 38,035 | 251,456 | 604,461 | 521 |
| Austria ...... | 1834-39 | 209,866 | 1 to 4 yrs. 80,830 | .. | 350,388 | 650,084 | 461 |
| Saxony ...... | 1832-41 | 16,746 | 1 to 6 yrs. 6,447 | .. | 23,227 | 46,420 | 499 |
| Frankfort-on-the-Maine. | 1840-42 | bth.to 5 | yrs. 357 | 25 | 754 | 1,136 | 314 |

A glance at these tables will suffice to show the very large relative mortality in infancy and childhood. It is well known that children are remarkably susceptible to all the injurious agents which tend to shorten life, and when such agents exist in full force, the mortality in early life becomes excessive. In Manchester, for instance, it is calculated that one-half of all the children die before they reach the age of five years. In healthy country districts the infantile mortality is much less. Of a thousand born in agricultural districts, two hundred and twenty-one will die under five years of age, showing a mortality less by half than that of Manchester. One-fourth of all the children born in England die before they reach their fifth birthday,—

"An unripe harvest for the scythe of death."

Here is the strongest possible argument for sanitary improvement, and for the diffusion among the mothers of England, of correct principles relating to the management of infancy and childhood.

The question next arises—what are the causes of this excessive mortality? They may be found under the following general divisions:—*First*. Original constitutional debility, or hereditary disease. *Secondly*. Acute diseases, such as measles, hooping-cough, scarlet fever, etc. *Thirdly*. Our general want of sanitary measures, giving rise to pollution of the atmosphere. *Fourthly*. Mismanagement with regard to diet and regimen in the nursery.

Of these four causes of mortality, the first three are common to all ages: the last alone is peculiar to infancy. The first is doubtless an item of some importance. Of the second it is to be observed, that the amount of infant mortality resulting from acute diseases might be greatly diminished by such a course of nursery government as would invigorate the constitutions of children. This is obvious. Every nurse knows that measles, or hooping-cough, proves fatal to the delicate child, but passes almost harmlessly by the robust. Respecting the third general cause I will remark, that the want of efficient sanitary measures, which presses so heavily on the health of the entire population, must obviously be especially injurious to the delicate frame of infancy; so much so that, as a general rule, we might take infantile mortality as a criterion of the sanitary condition of a neighbourhood. Thus, in some of the worst parts of London, one may find wretched men and women who, when questioned on their own health, will reply that they are "well" (though that is not true); but ask the women "where are their children," and one may probably extort such facts as that "one has only a solitary child left out of *seven*," and "*another has buried thirteen!*"*

But making a full allowance for the operation of these general causes, there must still be left a heavy balance of infant mortality to be ascribed, chiefly or solely, to mismanagement in the nursery.

To this last cause of infantile mortality I shall exclusively devote attention in the following articles. The rea-

* Godwin's London Shadows, p. 76.

son for thus giving it the priority is, that I regard it as surely and easily preventible; and the object is, to point out the two simple means of its prevention:—(1.) An increased attention to the subject on the part of medical men; (2.) Sound instructions for mothers and nurses. On the first of these preventive means I may venture to give a hint to my professional brethren; but my chief purpose is to assist in popularising the instructions that should be imparted to every mother and every nurse of children.

If we would diminish the excessive mortality of infancy, and prevent the numerous cases of physical misery in adults resulting from mismanagement *in the nursery*, we must not only popularise sound instruction of mothers and nurses, but must also urge the necessity for a greater degree of attention to the subject on the part of medical men, both lecturers and practitioners. The topics belonging to infantile hygiene should, it is conceived, form no inconsiderable part of every course of lectures on midwifery and diseases of children. Unfortunately, these topics, seeming so simple and easy that few will take the pains to study them, are, too often, passed over lightly and briefly. And what is the consequence? The young practitioner fresh from the schools, who has carefully studied the anatomy of the human body; who has studiously followed the surgeon round the wards of the hospital, and has constantly attended the operating theatre; who prides himself on his dexterity in amputating a limb in a few seconds, and waits impatiently for his first hernia operation—this practitioner, so far well educated, may, after all his studies, be puzzled when required to prescribe the best course of diet for a newly-born babe deprived of its natural source of nutriment! The medical man who could save life even in a rare case of surgical difficulty, may sacrifice the life of a healthy infant for want of knowledge of a few plain natural laws of diet and regimen!* Surely, here is an inconsistency

* We append one example of almost ludicrous mismanagement, which was happily corrected before it could reach a fatal crisis. After a lady's confinement, it was found advisable to use an embrocation to relieve difficulty of lactation. The precaution of washing the breast before attempting to suckle the infant was neglected! Very naturally, the infant, disgusted by the strong-smelling oils, refused to take the breast. This led to a second mistake—the determination to bring up the child by hand; and this was carried into effect in almost the worst possible way. The stomach was crammed with thick pap; cries of distress were mistaken for calls of hunger; and soon all the sufferings of infantile dyspepsia followed. When three months old, the child had wasted away to a mere skeleton, and weighed less than at its birth! At this stage it was rescued from mismanagement, and soon began to thrive.

that ought to be removed, as speedily as possible, from our plan of studies.

A well educated medical man should be prepared for any case that *may* occur in the course of his practice; but especially for cases that *must* demand his notice almost every day. It is requisite that he should know the remedy for a case of poisoning by aconite, and be able to treat successfully a compound fracture of the thigh-bone; but, in rural practice, years may pass away without a call for his aid in such cases, while he can scarcely pass through a village without finding some example of infancy suffering under mismanagement. It may seem, to an undisciplined mind, something low and beneath the dignity of the profession to give advice on the preparation of food for a babe,—to furnish recipes for such nursery dishes as panada, *crême de pain, lait de poule;* but it remains true that on such apparent trifles depend results far greater than those of many brilliant operations in surgery. Compare them. After the best exercise of skill in restoring to use the fractured limb in an adult or aged patient, what has been done? Probably ease and comfort have been secured for a few remaining years of life, and this is no slight boon. But by rescuing an infant from fatal mismanagement, a whole life—perhaps a long life—of vigour and physical happiness may be insured. To young students of medicine who are tempted to overlook homely and everyday duties, while exploring the subtleties of theory or rare cases in practice, we would commend the philosophy inculcated by Milton:—

> "Not to know at large of things remote
> From use, obscure and subtle, but *to know*
> *That which before us lies in daily life,*
> *Is the prime wisdom.*"

In the NURSERY, our attention to a few simple rules of diet and regimen may secure that basis of physical happiness, a vigorous constitution; or our errors may inflict injuries for which the utmost resources of medical science can supply no remedy. And so closely are mind and body united in this life, that even the moral character of the man may be affected by errors in the physical treatment of the infant. I might cite painful examples to support this assertion; but it may suffice here to refer to one—the abuse of alcoholic stimulants, sometimes administered to children by ignorant nurses. Of this more may be said in another place.

*(To be continued.)*

